# Imaging genotyping of functional signaling pathways in lung squamous cell carcinoma using a radiomics approach

**DOI:** 10.1038/s41598-018-21706-1

**Published:** 2018-02-19

**Authors:** So Hyeon Bak, Hyunjin Park, Ho Yun Lee, Youngwook Kim, Hyung-Lae Kim, Sin-Ho Jung, Hyeseung Kim, Jonghoon Kim, Keunchil Park

**Affiliations:** 1 Department of Radiology and Center for Imaging Science, Samsung Medical Center, Sungkyunkwan University School of Medicine, Seoul, Korea; 2 Department of Radiology, Kangwon National University Hospital, Chuncheon, Korea; 3 School of Electronic and Electrical Engineering, Sungkyunkwan University, Suwon, Korea; 4 Center for Neuroscience Imaging Research (CNIR), Institute for Basic Science, Suwon, Korea; 5 Samsung Advanced Institute for Health Sciences and Technology, Sungkyunkwan University School of Medicine, Seoul, Korea; 6 Department of Biochemistry, School of Medicine, Ewha Womans University, Seoul, Korea; 7 Statistics and Data Center, Research Institute for Future Medicine, Samsung Medical Center, Seoul, Korea; 8 Department of Electronic Electrical and Computer Engineering, Sungkyunkwan University, Suwon, Korea; 9 Division of Hematology/Oncology, Department of Medicine, Samsung Medical Center, Sungkyunkwan University School of Medicine, Seoul, Korea

## Abstract

Imaging features can be useful for identifying distinct genomic differences and have predictive power for certain phenotypes attributed to genomic mutations. We aimed to identify predictive imaging biomarkers that underpin genomic alterations and clinical outcomes in lung squamous cell carcinoma (SQCC) using a radiomics approach. In 57 patients with lung SQCC who underwent preoperative computed tomography (CT) and whole-exome DNA sequencing, 63 quantitative imaging features were extracted from CT and 73 clinicoradiological features including imaging features were classified into 8 categories: clinical, global, histogram-based, lung cancer-specific, shape, local, regional, and emphysema. Mutational profiles for core signaling pathways of lung SQCC were classified into five categories: redox stress, apoptosis, proliferation, differentiation, and chromatin remodelers. Range and right lung volume was significantly associated with alternation of apoptosis and proliferation pathway (p = 0.03, and p = 0.03). Energy was associated with the redox stress pathway (*p* = 0.06). None of the clinicoradiological features showed any significant association with the alteration of differentiation and chromatin remodelers pathway. This study showed that radiomic features indicating five different functional pathways of lung SQCC were different form one another. Radiomics approaches to lung SQCC have the potential to noninvasively predict alterations in core signaling pathways and clinical outcome.

## Introduction

Lung cancer has the highest mortality rate of all cancers and caused 1.59 million of the 8.2 million total cancer deaths in 2012^1,2^. In Korea, lung cancer has been the leading cause of cancer death since 1999, accounting for 22.8% of all cancer death in 2014^3^. In the last decades, there was dramatic paradigm shift in the management of advanced non-small cell lung cancer (NSCLC) with the identification of targetable driver mutations and development of molecularly targeted therapies. The progress, however, has been made largely in adenocarcinoma, not in squamous cell carcinoma^1,2,4^.

Lung squamous cell carcinoma (SQCC) accounts for 25–30% of NSCLC. SQCC is recognized as a distinct clinical and histological entity from other NSCLCs^1,5^. Compared with lung adenocarcinoma, actionable oncogene targets are rare in lung SQCC, limiting treatment options for advanced-stage lung SQCC with great unmet need^6^. Large, comprehensive analyses of lung SQCC cohorts showed that lung SQCC seem to be a different molecular entity from other lung cancer histological subtypes. These studies collectively identified genes that are altered preferentially in SQCC^5,7–9^.

Recent advances in understanding the molecular aberrations by comprehensive genotyping have led to the development of targeted agents for lung SQCC^2^. Targeted agents such as fibroblast growth factor receptor (*FGFR*) inhibitors, phosphatidylinositol 3-kinase (*PIK3K*) inhibitors, and insulin-like growth factor receptor 1 (*IGF1R*) monoclonal antibodies have been investigated in clinical trials based on molecular genotyping. However, most agents failed due to either toxicity or lack of efficacy^2,9^. Therefore, better understanding of tumorigenesis or effective targeting of molecular alterations of lung SQCC is necessary.

Imaging phenotypes potentially contain comprehensive information over genomics data^10,11^. Qualitative features such as tumor size or average parameter values usually used in routine practice or clinical radiology do not fully reflect the rich information content of tumors, and most of spatial information is discarded. On the other hand, quantitative imaging features based on radiomics have been expected to offer more unbiased and comprehensive information on tumor phenotypes and microenvironments^12,13^. We hypothesized that quantitative imaging features should be useful for identifying distinct genomic differences and have predictive power for phenotypes attributed to genomic mutations. Thus, the main aim of this study was to identify associations, if any, between imaging characteristics and alterations in major cancer genomic genes and pathways in lung SQCC and to identify potential predictive imaging biomarkers that underpin genomic alterations and clinical outcomes in lung SQCC using a radiomics approach.

## Results

### Demographic data

**Figure 1:**
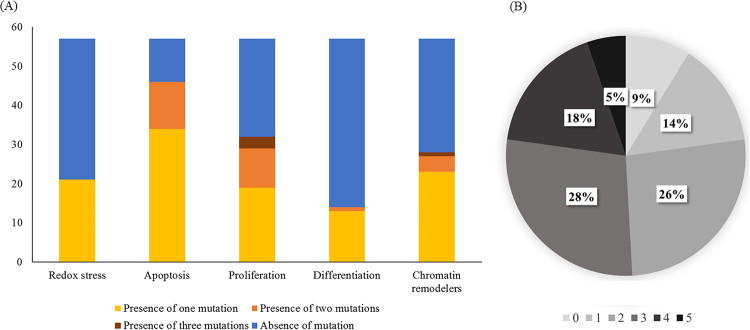
(**A**) Distribution of alteration percentage of signaling pathways, and (**B**) number of alteration of pathway per patients in lung SQCC.

This study included 57 patients (54 men, 3 women; mean age, 65.5 ± 6.7 years; range, 43.0–78.0 years): 3 never-smokers, 29 former smokers, and 25 current smokers; 22 stage I, 25 stage II, 8 stage III, and 2 stage IV. Patient characteristics are shown in Table 1. Lung SQCC is characterized by relatively high mutation rate (8.7 somatic mutation per Mb in the samples used in the study). However, at the gene level, even the most recurrently mutated top seven genes in the cohort, except the case of TP53, displayed the mutation percentage lower than 20% with most genes’ mutation rate less than 5% or non-existent. This paucity of mutations at the gene level causes practical difficulty in implementing detailed association study between somatic mutation of each gene and radiomic-features. Thus, we clustered the somatic mutations into five different functional categories, as suggested in the original genomic analysis of the cohort^8^. These five different functional groups include redox stress, apoptosis, proliferation, differentiation, and chromatin remodelers, each of which most comprehensively represents biological attributes of lung SQCC. Following mutations’ categorization into 5 representative lung SQCC pathways, it was assessed that 21 (36.8%) patients showed alternation of redox stress pathway, 46 (80.7%) in apoptosis pathway, 32 (56.1%) in proliferation pathway, 14 (24.6%) in differentiation pathway, and 28 (49.1%) in chromatic remodelers pathways. Most patients had multiple alterations of cancer pathways (Fig. 1). Most patients (77%) with alteration pathways had aberration of one of the genes associated with the pathway. Patients with alteration of the apoptosis and proliferation pathways had two gene aberrations simultaneously in 12 and 10 patients, respectively. Figure 1 shows the distribution of alteration of functional signaling pathways in lung SQCC.

**Table 1:** Demographics of 57 patients with lung squamous cell carcinoma.

Variables	No.	%
Age*	65.5 ± 6.7	43.0–78.0
Sex		
Male	54	94.7
Female	3	5.3
Smoking state		
Non-smoker	3	5.3
Ex-smoker	29	50.9
Current smoker	25	43.8
TNM stage		
I	22	38.6
II	25	43.8
III	8	14
IV	2	3.5
T descriptor		
T1	10	17.5
T2	37	64.9
T3	8	14
T4	2	3.5
N descriptor		
N1	41	71.9
N2	9	15.8
N3	7	12.3
M descriptor		
M0	55	96.5
M1	2	3.5
Treatment		
Adjuvant chemotherapy or radiation therapy	13	22.8
Palliative chemotherapy or radiation therapy	10	17.4
Operation for recurrence or metastasis	4	7.0
Survival		
Death	18	31.6
Disease-free survival (months)*	44.5 ± 31.5	
Overall survival (months)*	51.4 ± 30.2	

*Data are mean ± SD and range.

### Imaging genotyping of functional signaling pathway

This study investigated to quantitative imaging biomarkers that can predict the alteration of five biological pathways in lung SQCC using univariate and multivariate logistic analyses. Selected variables associated with alteration of five functional pathways are summarized in Table 2. A transcription factor pathway that regulates redox stress was altered in 21 patients (36.8%) (Fig. 1). Volume (global), energy (histogram-based), maximum 3D diameter (shape), cluster prominence (local), and size-zone variability (regional) were selected as variables associated with alteration of the redox stress pathway. Of the selected features, energy had the strongest correlation tendency with pathway alteration, although the correlation was not significant (*p* = 0.06; odds ratio, 0.15; 95% CI, 0.02–1.05). For predicting alteration of the redox stress pathway, the area under the receiver operating characteristic (ROC) curve (AUC) was 0.698 when selected variables were combined.

**Table 2:** Selected features for prediction of targetable pathways.

Pathway		Clinical features	Global variables	Histogram-based features	Lung cancer-specific features	Shape features	Local features	Regional features	Emphysema features
Redox stress	Variables		Volume	Energy		Maximum 3D diameter	Cluster prominence	Size-zone variability	
	Odds ratio (95% CI)		1.64 (0.32–8.30)	0.15 (0.02–1.05)		0.88 (0.20–3.82)	1.28 (0.64–2.56)	2.16 (0.66–7.06)	
	*p* value		0.55	0.06		0.86	0.49	0.20	
Apoptosis	Variables		Mass	**Range**		Maximum 3D diameter	Cluster shade	Intensity variability	
	Odds ratio (95% CI)		23.94 (0.54- > 999.99)	0.08 (0.01–0.82)		2.06 (0.16–26.46)	0.69 (0.13–3.80)	0.35 (0.06–2.26)	
	*p* value		0.10	0.03		0.58	0.67	0.27	
Proliferation	Variables			HU at the 97.5th percentile	MPP		Cluster prominence		**Right lung volume**
	Odds ratio (95% CI)			1.83 (0.69–8.61)	1.33 (0.23–7.56)		1.77 (0.82–3.81)		0.35 (0.13–0.90)
	*p* value			0.44	0.75		0.15		0.03
Differentiation	Variables	Smoking pack-years	Minimum			Spherical disproportion		Intensity variability	
	Odds ratio (95% CI)	1.01 (0.99–1.04)	1.21 (0.59–2.47)			0.68 (0.33–1.43)		0.73 (0.32–1.68)	
	*p* value	0.26	0.60			0.31		0.46	
Chromatin remodelers	Variables	Smoking pack-years	Energy				Maximum probability		
	Odds ratio (95% CI)	1.02 (0.99–1.04)	1.73 (0.94–3.19)				0.54 (0.28–1.05)		
	*p* value	0.11	0.08				0.07		

Data are features selected as variables using univariate analysis by category.

Bold are features selected as independent predictive factors on multivariate analysis.

CI, confidence limits; MPP, mean value of positive pixe.

An apoptosis pathway was frequently altered (80.7%), and mass (global), range (histogram-based), maximum 3D diameter (shape), cluster shade (local) and intensity variability (regional) were selected as the variables associated. Range was significantly associated with alteration of the apoptosis pathway on multivariate analysis (*p* = 0.03; odds ratio, 0.08; 95% CI, 0.01–0.82). The AUC was 0.868 for prediction of alteration in the apoptosis pathway.

A pathway regulating proliferation was altered in 32 patients (56.1%). Features selected as variables were Hounsfield unit (HU) at the 97.5th percentile (histogram-based), mean value of positive pixels (MPP, lung cancer-specific), cluster prominence (local), and right lung volume (emphysema). Right lung volume was significantly associated with alteration of proliferation pathway on multivariate analysis (*p* = 0.03; odds ratio, 0.35; 95% CI, 0.13–0.90). The AUC was 0.812 as for prediction of alteration in the proliferation pathway.

A squamous cell differentiation pathway was altered in 14 patients (24.6%). The variables associated with alteration of the differentiation pathway were smoking pack-years (clinical), minimum (histogram-based), spherical disproportion (shape), and intensity variability (regional). The AUC was 0.684 for prediction of alteration of the differentiation pathway. A chromatin remodeler pathway was altered in 28 patients (49.1%). The variables associated with alteration of the chromatin remodeler pathway were smoking pack-years (clinical), energy (histogram-based), and maximum probability (local). The AUC was 0.710 for prediction of alterations in the pathway. No clinicoradiological features were significantly associated with the alteration of differentiation and chromatin remodeler pathways on multivariate analysis.

Most of the patients included in this study were males (94.7%), and further analysis of the association between smoking and alteration of signaling pathway was performed. However, the state or pack-years of smoking and alteration of signaling pathways were not statistically significant (*p* > 0.05, Supplementary Figure 1).

### Survival

After surgical resection of the SQCC, 13 (22.8%) of 57 patients underwent adjuvant treatment, 10 (17.5%) underwent palliative chemotherapy or radiation therapy, and 4 (7.0%) underwent metastasectomy. Nineteen (33.3%) of 57 patients experienced local tumor recurrence or metastasis. We investigated whether a relationship exists between clinicoradiological features and disease-free survival (DFS) or overall survival (OS). Using the Cox regression model, N descriptor [*p* = 0.02, hazard ratio (HR), 0.19; 95% CI, 0.05–0.79], kurtosis (*p* = 0.02, HR, 7.52; 95% CI, 1.43–39.57), surface area (*p* = 0.02, HR, 14517.77; 95% CI, 5.71–36926015.07), and spherical distortion (*p* = 0.04, HR, 0.00; 95% CI, 0.00–0.03) were significantly associated with DFS. ROC curves measured the performance of a prediction model about DFS, and the AUC was 0.866. In terms of overall survival, age (*p* = 0.04, HR, 1.09; 95% CI, 1.01–1.19), T descriptor (*p* = 0.01, HR, 3.36; 95% CI, 1.41–7.99), interquartile range (IQR) (*p* = 0.01, HR, 0.01; 95% CI, 0.00–0.33), HU at the 25th percentile (*p* = 0.01, HR, 0.01; 95% CI, 0.00–0.27), and HU at the 97.5th percentile (*p* = 0.00, HR, 3.88; 95% CI, 1.86–8.14) were significantly associated with overall survival. The AUC for prediction of OS was 0.674.

## Discussion

Rapid advances in our understanding of the molecular pathogenesis that underlies lung cancer have altered diagnostic algorithms and led to the identification of molecular targets for treatment^14,15^. Information about tumor genotypes is usually evaluated with small amount of tissue extracted through biopsy from the particular part of a whole tumor showing intratumor clonal heterogeneity^16^. Therefore, the genetic information obtained from a small amount of tumor biopsy may not represent that of the whole cancer^17^, particularly for subclonal mutations occurring later during cancer evolution^18^. In contrast, imaging is more representative of the whole tumors and may facilitate identification of mutations and have a clinical influence on precision medicine^19,20^. Therefore, we aimed to identify potential clinicoradiological candidates to predict alterations in representative cancer pathways and clinical outcome in lung SQCC.

Medical imaging is useful for noninvasively assessing the characteristics of tumor tissue^21^. In lung adenocarcinoma with well-known, targetable mutations, studies have found imaging biomarkers that reflect gene expression or treatment response^17,19,22–25^. However, lung SQCC has no well-known, targetable gene mutations, and clinical trials of target agents have not been successful for lung SQCC compared to lung adenocarcinoma^2,9^. Given that oncogenic driver mutations and functional signaling pathways of lung SQCC have been identified^7,8,26^, correlation of imaging features with specific gene expression could help identify specific imaging phenotypes related to functional behavior or prognosis^10^.

Our previous study described that lung SQCCs showed a high mutational burden in lungs SQCCs in a large cohort of East Asians, and statistical enrichment for mutations in 7 genes; *TP53*, *RB1*, *PTEN*, *NFE2L2*, *KEAP1*, *MLL2*, and *PIK3CA*^8^. Building on a previous study, we conducted a study to find clinicoradiological features that could predict alterations in representative cancer pathways by analyzing 57 patients with lung SQCC who underwent preoperative CT and whole-exome sequencing. To the best of our knowledge, this is the first study to evaluate relationships between imaging features and alterations in genes and/or functional cancer pathways in lung SQCC.

Heterogeneity is a common feature of malignancies containing areas of high cell density, necrosis, hemorrhage, and myxoid change^27^. Genetic heterogeneity of a malignant tumor leads to regional differences in stromal architecture or spatial heterogeneity and could consequently be illustrated as imaging phenotypes^13^. Radiomics is an emerging and robust field that extracts quantitative features from images using computer algorithms^11,21^. Quantitative imaging features have the potential to noninvasively convey comprehensive intra-tumor, inter-tumor and peri-tumor information^28^. Most prominently, 1^st^ order, histogram-based radiomic features represent information about the distribution of voxel intensities within tumor on CT image and have been popular for characterizing intra-tumoral heterogeneity^13,21^. Likely, our study showed a correlation between histogram-based features of range, energy, and HU at the 97.5th percentile with alterations in apoptosis, redox stress, and proliferation pathways. Meanwhile, higher order, texture features extracted from gray level co-occurrence (GLCM) matrix reflect the textural characteristics of tumors, retaining spatial information among pixels within tumor on CT images^29^. In adenocarcinoma, local features were found to be associated with mutations in genes such as epidermal growth factor receptor (*EGFR*) or anaplastic lymphoma kinase (*ALK*)^19,25^. In the present study in lung SQCC, local features such as cluster prominence, cluster shade, and maximum probability were associated with alteration of pathway. Ganeshan *et al*.^27^ showed that MPP has the potential to act as an imaging correlate for tumor hypoxia and angiogenesis. In our study, MPP was associated with a proliferation pathway, which is relevant because MPP is a value to deal with only positive pixels representing invasive component. More importantly and interestingly enough, radiomic features indicating five different functional pathways of lung SQCC were different from one another.

Two large, prospective cohort studies showed that the presence of emphysema on CT is an independent risk factor of lung cancer^30,31^. Smith *et al*.^32^ demonstrated that the presence of emphysema on CT is independently associated with a specific histological subtype of NSCLC termed SQCC. Lung cancer and chronic obstructive pulmonary disease share a common risk factor of cigarette smoking and should be considered to share similar pathogenic mechanisms^33^. Consequently, we hypothesized that alterations in lung SQCC cancer pathways may be associated with emphysema on CT. However, we failed to show a significant association between alteration of functional signaling pathways and emphysema. The biological meaning of statistical association between right lung volume and proliferation pathway remains unclear and needs to be validated and further investigated. Allowing for limited number of our study, further studies with larger populations are necessary to clarify their relationship at a more refined level, including individual genes.

Compared with lung adenocarcinoma, few effective therapeutic advances have been made for lung SQCC patients. This has contributed to poor outcomes and a high mortality rate for lung SQCC^1,34^, for which advanced prognostic stratification regarding SQCC would be useful to select more appropriate candidate who needs more aggressive treatment. Kinoshita *et al*.^35^ reported that high serum levels of squamous cell carcinoma antigen and pleural and vascular invasions are independent prognostic factors of completely resected peripheral SQCC. On the other hand, prognostic imaging biomarkers for lung SQCC patients have not been established, not even using a radiomics approach. In our study, shape-based features such as surface area and spherical disproportion are the features associated with DFS. Given that surface area and spherical disproportion are values reflecting the irregularity of the tumor shape, degree of non-uniform growth of tumor would be considered to be significant negative prognostic factor for SQCC. A study with lung adenocarcinoma reported that the irregular shape of tumor was associated with worse survival, similar to our results^36^. When it comes to prognostic imaging biomarker for OS in the present study, histogram-based features such as IQR, HU at the 25^th^ and 97.5^th^ percentile showed the independent prognostic potential in patients with SQCC. A histogram shows the range and frequency of pixel values within the defined region of interest (ROI), and IQR is a value indicating variability, based on dividing a histogram into quartiles indicating variability^29,37^. Therefore, the value of IQR reflect the heterogeneously distributed tissues, and intratumoral heterogeneity of tumor is correlated with OS in patients with SQCC. Emphysema is commonly found in patients with lung SQCC^38^ because both of them share smoking history as a risk factor. Emphysema demonstrated the association with unfavorable prognosis in the patients with SQCC in several studies^38,39^. However, our study excluded emphysema from the survival analysis because there were some cases that failed to obtain quantitative image features representing the degree of emphysema.

Our study had several limitations. First, since the patient number was small, our results did not have sufficient statistical power. In this study, we tried to perform validation such as 10-fold validation, however, a small number of patients was limited to validation. Second, not a prospective study, therefore, the results are not conclusive and require validation by prospective studies in a larger cohort. However, our study dealing with in depth radiomics and genome-level sequencing of tumor DNA is invaluable considering that creating larger cohorts of patients with complete genome-level sequencing data would be logistically difficult. In addition, we did not consider variability of image acquisition and reconstruction because this study dealt with homogenous imaging protocol, conducted at a single center.

## Conclusion

Radiomics approaches may have the potential to act as imaging biomarkers correlates for functional pathways of lung SQCC. In our study, different radiomics features reflected the aberration of each functional pathway. Shaped-based and histogram-based features were associated with reduced survival, also. Quantitative imaging biomarkers would allow a comprehensive evaluation of the molecular status and functional signaling pathways of lung SQCC and lead to development of more effective target agents with less toxicity.

## Methods

### Dataset

This retrospective study conducted at a single tertiary cancer center was approved by the Institutional Review Board of Samsung Medical Center, and informed consent was waived. We included 57 patients with lung SQCC from 2005 to 2012. Inclusion criteria were: (1) undergoing surgical resection, (2) no neoadjuvant therapy prior to surgery, (3) available genomic data, and (4) preoperative CT for diagnosis and quantitative image analysis. We retrospectively reviewed medical records for clinical characteristics, clinical outcomes, gene mutation, and preoperative chest CT.

### Biospecimen Collection and Mutational Analyses

**Figure 2:**
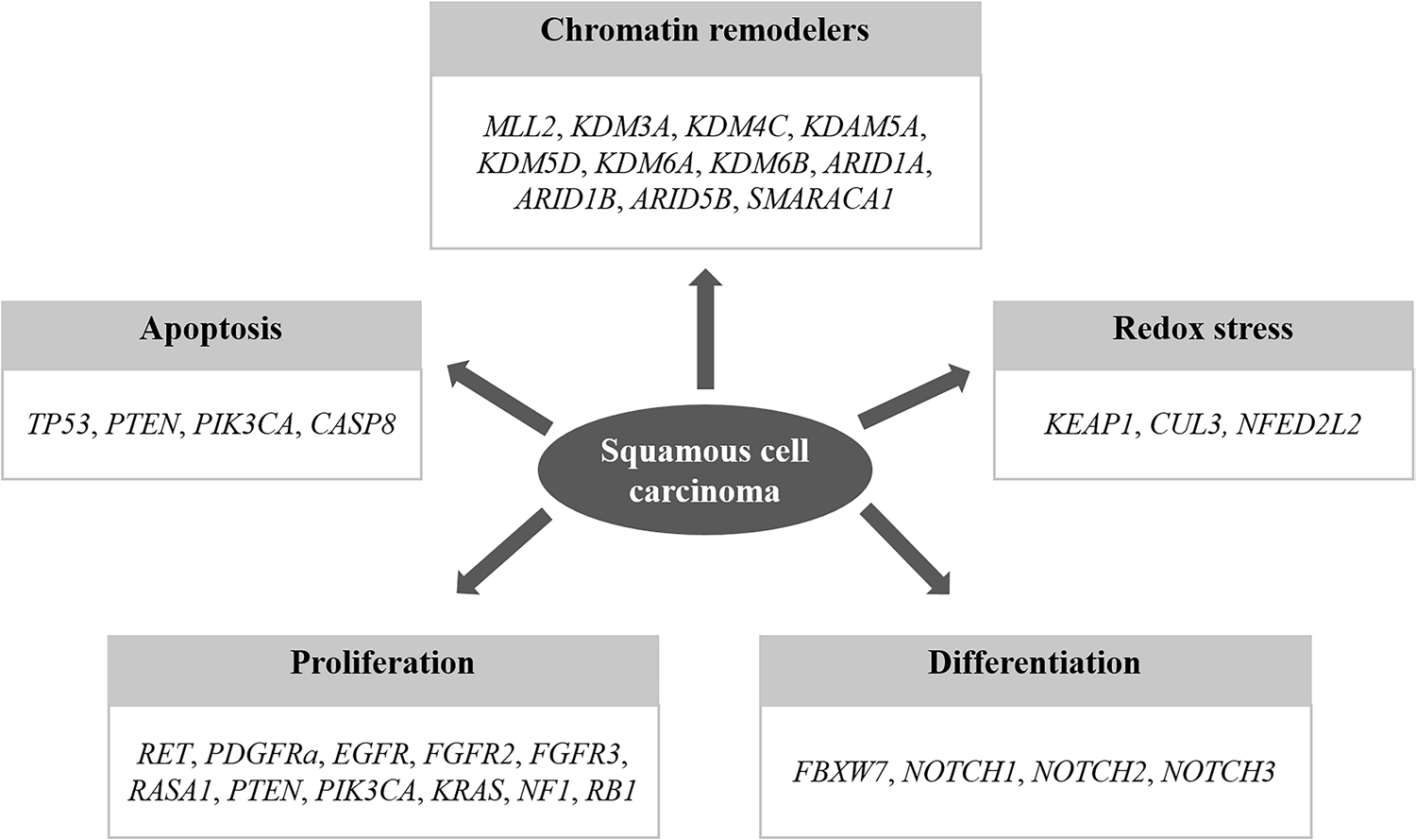
Five functional signaling pathways and genes used for analysis of lung SQCC (Kim *et al*. J Clin Oncol 2013)^8^.

Analysis of sequencing data was as previously described^7,40^. Tissue samples of 57 lung SQCC were collected with matched adjacent normal tissues. Whole-exome sequencing was based on the amount of extracted DNA. DNA sequencing and data processing were as in a previous study^8^. Sequencing was conducted by Macrogen, Inc. (Seoul, Korea). Basic alignment and sequencing quality control was performed on Picard and Firehose pipelines at the Broad Institute and on in-house pipelines at Macrogen, Inc. (Seoul, Korea) and Theragen, Bio (Suwon, Korea). Sequencing data were processed following known protocols^7,40–42^. ‘Picard’ uses the reads and qualities from Illumina software to produce a single BAM file (http://samtools.sourceforge.net/SAM1.pdf). BAM files for matched tumor and normal samples were further processed and analyzed in the cancer genome analysis pipeline Firehose. Components include ContEst, Mutect, Indelocator, and dRanger^40,43–45^. Significance of mutations was calculated using the MutSig algorithm^40,42,46^ and implemented as described previously^7^. The outline of the entire pipeline can be accessed at www.broadinstitute.org/cancer/cga. Mutational profiles of five different signaling pathways of lung SQCC were used in analysis^8^ (Fig. 2). Alteration of signaling pathways was defined as the presence of aberration in more than one of the genes associated with the pathway.

### Image Acquisition

Chest CT scans were performed before surgery. All helical CT images were obtained using a 64 detector-row (LightSpeed VCT; GE Healthcare, Waukesha, WI) CT scanner using the following parameters: 125 mA; 120 kVp; beam width, 10–20 mm; beam pitch, 1.375–1.5. Image data were reconstructed with a section thickness of 2.5 to 5 mm. Scan field of view used were large body. Image data were reconstructed with a soft-tissue algorithm for mediastinal window ranges and a bone algorithm for lung window images. Detailed methods for CT scans are described in Supplementary Appendix 1.

### Image Analysis

**Figure 3:**
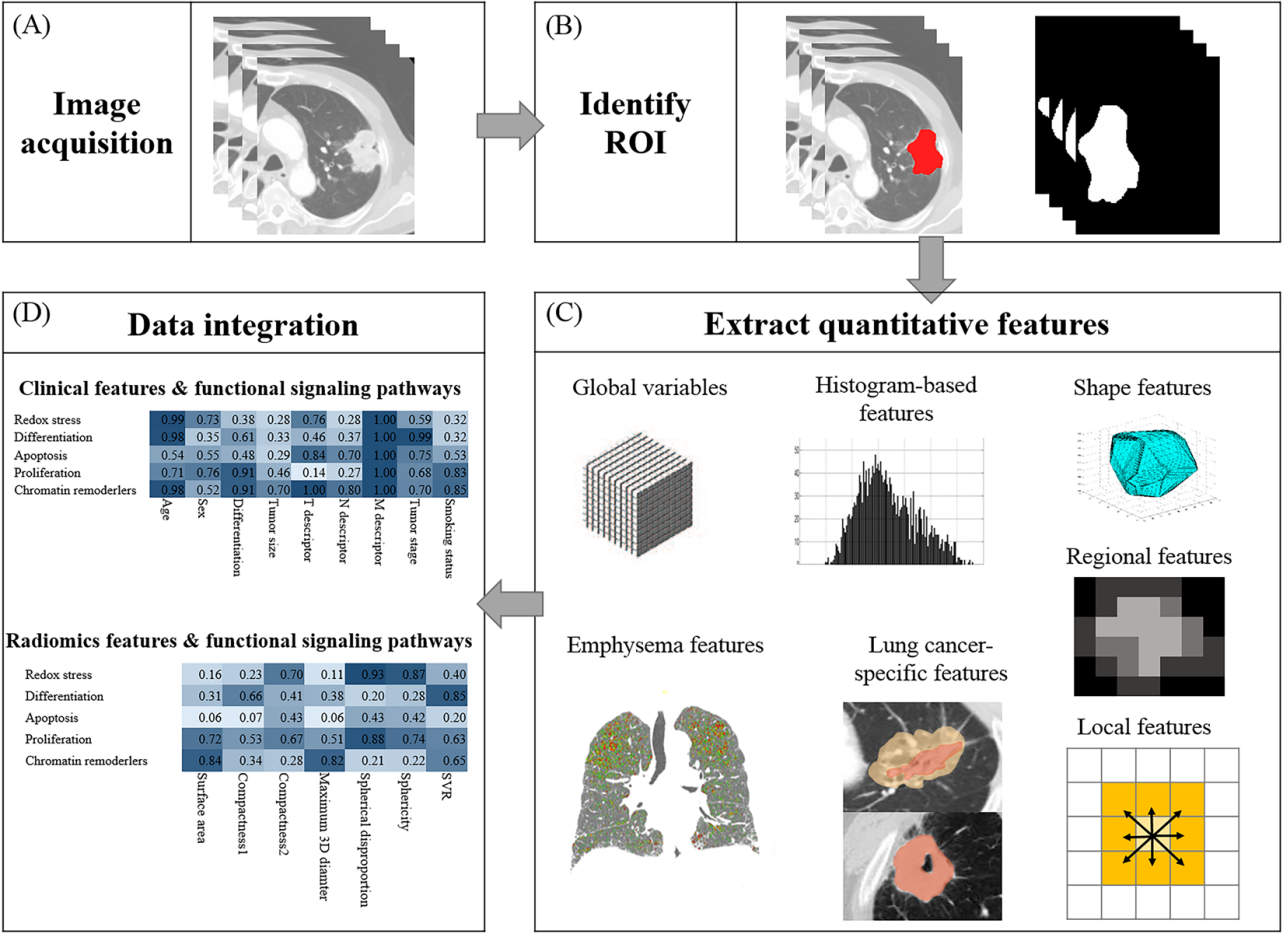
Extracting quantitative imaging features from CT images. (**A**) CT images were obtained on full inspiration. (**B**) Tumors were segmented by drawing regions of interest (ROIs) that traced tumor edges on all axial images. (**C**) Quantitative image features were extracted from within defined tumor contours on CT images to quantify features that were global, histogram-based, shape, lung cancer-specific, local, regional, or emphysema. (**D**) Associations among quantitative image features, clinical data, and gene expression data were analyzed.

A total of 52 quantitative features were computed using in-house MATLAB code (Mathworks Inc., MA, USA). Features were divided into six categories: 1) global, 2) histogram-based, 3) lung cancer-specific, 4) shape-based, 5) local, and 6) regional category. All features were computed over a manually drawn ROI by a radiologist using MRIcro (version 1.40, Chris Rorden, University of Nottingham, Great Britain). Tumors were segmented by drawing an ROI that traced the edge of the tumor on all axial images until the entire tumor was covered (Fig. 3). Each feature quantified a different aspect of the tumors. For instance, shape-based features reflected morphological information. Details of the adopted features are described in Supplementary Appendix 2 and Supplementary Table 1.

A total of 11 emphysema features for lung parenchyma were obtained using a workstation (Thoracic-VACR, GE healthcare). CT scans with section thickness more than 2.5 mm were excluded from volumetric analysis (n = 15). Emphysema analysis was conducted for 42 patients. For emphysema analysis, parameters obtained on full inspiratory CT were total lung volume, right and left lung volume, emphysema volume, emphysema index, normal lung volume, and normal lung percentage. Lung segmentation was performed semiautomatically. Emphysema was defined as lung pixels with attenuation of −950 HU or less on inspiration CT. Normal lung volume was defined by subtraction of emphysema volume from total or right/left lung volume^47^. A total of 73 clinicoradiological features were placed into 8 categories (Supplementary Table 2).

### Statistical Analysis

Clinical and quantitative image features were used to identify biomarkers of altered signaling pathways or survival. Univariate and multivariate logistic analyses were used to identify associations between alteration of lung SQCC pathways and clinicoradiological features. The *p* values of 73 features were calculated using univariate logistic analysis. In each category, features with the smallest *p* value were selected as biomarkers (Supplementary Table 3). If no feature had a *p* value less than 0.2, we did not select features in this category. Features significant for predicting the alteration of functional signaling pathways and survival were selected using multivariate analysis. The association between the smoking state or pack-year and alteration of functional signaling pathway was analyzed using Fisher’s exact test and Wilcoxon rank sum test. PFS and OS were defined as the time from the operation to recurrence (for PFS)/death event (for OS) or the time to last follow up. Cox regression analysis was used to identify association between survival and clinicoradiological features. Associations between selected features and aberrations in pathways or survival were evaluated by AUC for the presence or absence of pathway alteration and survival. Analyses were performed using SAS version 9.4 (SAS institute, Cary, NC) and R 3.3.1 (Vienna, Austria; http://www.R-project.org/). A *p* value less than 0.05 was considered statistically significant.

## Electronic supplementary material

Supplementary information
