# Supplementary material for: Imaging genotyping of functional signaling pathways in lung squamous cell carcinoma using a radiomics approach
Source: Sci Rep. 2018 Feb 19;8:3284. doi: 10.1038/s41598-018-21706-1 (PMC5818618; doi:10.1038/s41598-018-21706-1)
Supplement: Supplementary file 1 — Supplementary information [file 41598_2018_21706_MOESM1_ESM.pdf]

Supplementary Information

Imaging genotyping of functional signaling pathways in lung squamous cell carcinoma using a radiomics approach

So Hyeon Bak[1,2+], Hyunjin Park[3,4+], Ho Yun Lee[1*], Youngwook Kim[5], Hyung-Lae Kim[6], Sin-Ho Jung[7], Hyeseung Kim[7], Jonghoon Kim[8], Keunchil Park[9*]

[1]Department of Radiology and Center for Imaging Science, Samsung Medical Center, Sungkyunkwan University School of Medicine, Seoul, Korea

[2]Department of Radiology, Kangwon National University Hospital, Chuncheon, Korea

[3]School of Electronic and Electrical Engineering, Sungkyunkwan University, Suwon, Korea

[4]Center for Neuroscience Imaging Research (CNIR), Institute for Basic Science, Suwon, Korea

[5] Samsung Advanced Institute for Health Sciences and Technology, Sungkyunkwan University School of Medicine, Seoul, Korea

[6]Department of biochemistry, School of Medicine, Ewha Womans University, Seoul, Korea

[7]Statistics and Data Center, Research Institute for Future Medicine, Samsung Medical Center, Seoul, Korea

[8]Department of Electronic Electrical and Computer Engineering, Sungkyunkwan University, Suwon, Korea

[9]Division of Hematology/Oncology, Department of Medicine, Samsung Medical Center, Sungkyunkwan University School of Medicine, Seoul, Korea

+These authors contributed equally to this study.

*Correspondence to:

Ho Yun Lee, MD, Ph.D.

Department of Radiology and Center for Imaging Science, Samsung Medical Center, Sungkyunkwan University School of Medicine, 81 Irwon-ro, Gangnam-gu, Seoul 135-710, Korea

E-mail: hoyunlee96@gmail.com

Keunchil Park, MD, PhD.

Division of Hematology/Oncology, Department of Medicine, Samsung Medical Center, Sungkyunkwan University School of Medicine, 81 Irwon-ro, Gangnam-gu, Seoul 135-710, Korea

E-mail: kpark@skku.edu

Supplementary Material and Methods

Supplementary Appendix 1

Chest CT scans were obtained from lung apices to the level of the middle portion of both kidneys immediately after intravenous contrast medium injection. A total of 1.5 mL/kg (body weight) Iomeron 300 (Iomeprol, 300 mg iodine/mL; Bracco; Milan, Italy) was injected at an infusion rate of 3 mL/s using a power injector (MCT Plus; Medrad; Pittsburgh, PA, USA). Both mediastinal (width, 400 Hounsfield units [HU]; level, 20 HU) and lung (width, 1500 HU; level -700 HU) window images were displayed for tumor assessment. Reconstructed images were retrieved using the Picture Archiving and Communications System (PACS) (Centricity 2.0, GE Healthcare, Mt. Prospect, IL, USA).

Supplementary Appendix 2

We adopted radiomics features to extract multi-dimensional information of a given region of interest (ROI)[1]. Histogram-based features were computed using intensity (HU) distribution of a given ROI. These features reflected intensity characteristics of the ROI. They were simple measurements that have been widely adopted as standard features to quantify tumors. We computed 16 features covering energy and range[2]. Shape-based features reflected morphological information of an ROI. We computed 7 features covering surface area, sphericity, and compactness[1]. Local features or gray level co-occurrence (GLCM)-related features consider intensity values of a neighborhood instead of a single voxel. Similarity and dissimilarity of voxel intensities within a neighborhood can be quantified. Thus, GLCM-related features quantify texture information[3]. GLCM features were computed for an immediate voxel

neighborhood over 13 directions in 3D. A total of 20 features including auto-correlation and cluster shade were computed[1]. Intensity size zone (ISZ) features are also related to texture but can quantify beyond the immediate neighborhood. The feature assumes that an ROI can be further divided into subregions with uniform intensity and variable size. ISZ quantifies the number of subregions and how often certain subregions occur within the tumor[4]. A total of 2 features, intensity variability and size-zone variability, were computed.

References

1 Aerts, H. J. *et al.* Decoding tumour phenotype by noninvasive imaging using a quantitative radiomics approach. *Nature communications* **5**, 4006, doi:10.1038/ncomms5006 (2014).

2 Davnall, F. *et al.* Assessment of tumor heterogeneity: an emerging imaging tool for clinical practice? *Insights into imaging* **3**, 573-589, doi:10.1007/s13244-012-0196-6 (2012).

3 Zinn, P. O. *et al.* Radiogenomic mapping of edema/cellular invasion MRI-phenotypes in glioblastoma multiforme. *PloS one* **6**, e25451, doi:10.1371/journal.pone.0025451 (2011).

4 Tixier, F. *et al.* Intratumor heterogeneity characterized by textural features on baseline 18F-FDG PET images predicts response to concomitant radiochemotherapy in esophageal cancer. *Journal of nuclear medicine : official publication, Society of Nuclear Medicine* **52**, 369-378, doi:10.2967/jnumed.110.082404 (2011).

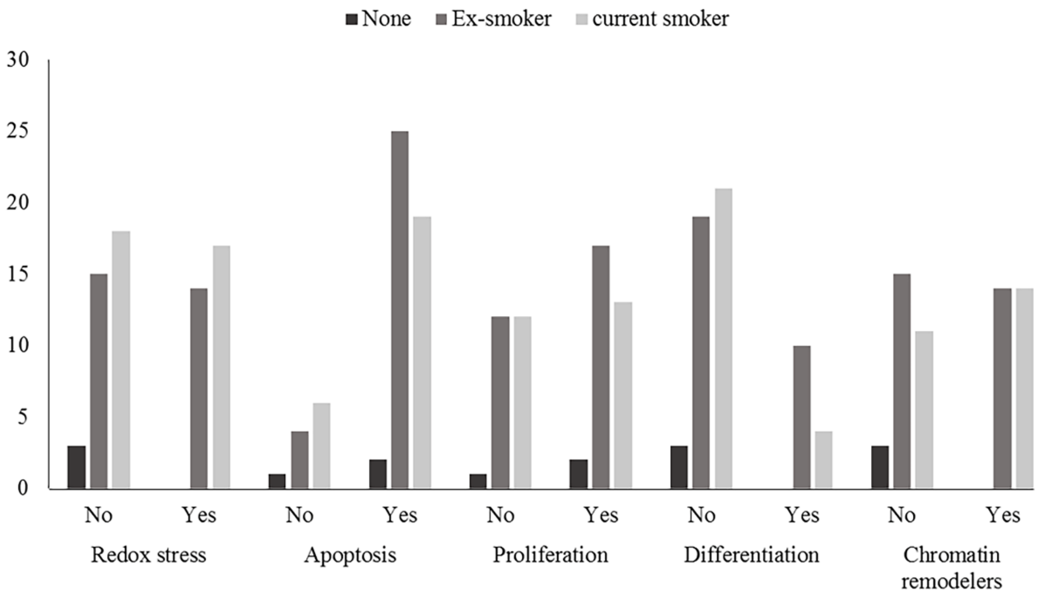

Supplementary Figure 1. Distribution of alteration of functional signaling pathways according to smoking status.

Supplementary Table 1: Quantitative CT features

	Parameter	Formula	Description
**Global variables**	Volume	$\text{Volume} = R * \text{number of voxels}$<br>where R denotes the 3D image resolution	Volume of tumor (ROI)
	Mass	$\text{Mass} = V * D$<br>where V denotes the tumor volume, D denotes the tumor density	Mass of tumor (ROI)
	Density	$\text{Density} = \frac{M}{V}$<br>Where V denote the tumor volume, M denote the tumor mass	Density of tumor (ROI)
	Mean	$\text{Mean} = \frac{1}{N}\sum_{i}^{N} X(i)$<br>Where X denote the 3d image matrix with N voxel.	Measures mean intensity value of a histogram
	Standard deviation	$\text{Std} = \left(\frac{1}{N-1}\sum_{i=1}^{N} (X(i) - \bar{x})^2\right)^{1/2}$<br>Where X denote the 3d image matrix with N voxel.	Measures amount of variation of a histogram.
**First-order features** (Histogram-based features)	Variance	$\text{Variance} = \frac{1}{N-1}\sum_{i=1}^{N} (X(i) - \bar{x})^2$	Measures squared distances of each value of a histogram from the mean
	Maximum	$\text{Max} = \max(X(i))$<br>Where X denote the 3d image matrix	Measures maximum intensity value of a histogram
	Minimum	$\text{Min} = \min(X(i))$<br>Where X denote the 3d image matrix	Measures minimum intensity value of a histogram
	Interquartile range (IQR)	$\text{IQR} = Q_3 - Q_1$<br>Where Q_3 denote the 3rd quartile of histogram, Q_1 denote the 1st quartile of histogram	Measures of variability, based on dividing a histogram into quartiles

	Range	$\text{Range} = \text{range}(X(i))$	Measures the difference between the highest and lowest voxel values of a histogram
	Root mean square (RMS)	$\text{RMS} = \sqrt{\frac{1}{N}\sum_{n=1}^{N}\lvert X_n\rvert^2}$<br>where X denotes the 3d image matrix with N voxels	Measures the square-root of the mean of the squares of the values of the histogram. This feature is another measure of the magnitude of a histogram
	Skewness	$\text{Skewness} = \frac{E(X-\mu)^3}{\sigma^3}$<br>where μ denotes the mean of X, σ denotes the standard deviation of x, E is the expectation operator.	Measures asymmetry of a histogram
	Kurtosis	$\text{Kurtosis} = \frac{E(X-\mu)^4}{\sigma^4}$<br>where μ denotes mean of X, σ denotes standard deviation of x, E is the expectation operator.	Measures "peakedeness" of a histogram (flatness of histogram)
	Energy	$\text{Energy} = \sum_{i}^{N} X(i)^2$<br>where X denotes the 3d image matrix with N voxels.	Measures squared magnitude value of a histogram
	Entropy	$\text{Entropy} = -\sum_{i=1}^{N_l} P(i)\log_2 P(i)$<br>where P denotes the first-order histogram with N_l discrete intensity levels	Measures irregularity of a histogram
	Uniformity	$\text{Uniformity} = \sum_{i=1}^{N_l} P(i)^2$<br>where P denotes the first-order histogram with N_l discrete intensity levels	Measures uniformity of a histogram
	Histogram percentile	$\text{Percentile} = \left(\frac{n^{th}\ \text{percentile}}{100}\right) * X(i)$	Measures intensity value at the 2.5^{th},25th,50th,75th, and 97.5^{th} percentile on histogram
**Lung cancer-specific features**	Mean value of positive pixels (MPP)	$\text{MPP} = \frac{1}{N_+}\sum_{i}^{N} X(i)$<br>where N_+ denotes total number of positive gray level pixels in $X(i)$	Measures average of positive value of a histogram
	Uniformity value of positive pixels (UPP)	$\text{UPP} = \sum_{i=1}^{N_l} \lvert P(i)\rvert^2$<br>where P denotes the first order histogram with N_l discrete intensity levels	Measures uniformity of positive values of a historgam
**Shape-based features**	Compactness1	$\text{Compactness1} = \frac{V}{\sqrt{\pi}A^{\frac{2}{3}}}$<br>where V denotes the volume, and A denotes the surface area of the volume of interest (VOI)	Quantifies how close an object to the smoothest shape, the circle

	Surface area	$\text{SA} = \sum_{i=1}^{N} \frac{1}{2} \lvert a_i b_i \times a_i c_i \rvert$ where N denotes the total number triangle (covered surface area), and a, b, c are edge vectors	The surface area of the ROI
	Sphericity	$\text{Sphericity} = \frac{\pi^{\frac{1}{3}} \times (6V)^{\frac{2}{3}}}{A}$ where A denotes area, and V denotes volume	Measures of the roundness of the ROI
	Spherical disproportion	$\text{Spherical disproportion} = \frac{A}{4\pi R^2}$ where R denotes the radius of a sphere with the same volume as the tumor	The ratio of the surface area of the ROI to the surface area of a sphere with the same volume as the ROI
	Surface-to-volume ratio (SVR)	$\text{SVR} = \frac{A}{V}$ where A denotes area, and V denotes volume	Surface to volume ratio
**Gray level co-occurrence (GLCM, local) features**	Autocorrelation	$\text{Autocorrelation} = \sum_{i=1}^{N_g} \sum_{j=1}^{N_g} ij\mathbf{P}(i,j)$	Measures of the magnitude of the fineness and coarseness of texture
	Cluster Prominence	$\text{Cluster prominence} = \sum_{i=1}^{N_g} \sum_{j=1}^{N_g} [i + j - \mu_x(i) - \mu_y(j)]^4 \mathbf{P}(i,j)$	Measures number of potential clusters present
	Cluster shade	$\text{Cluster shade} = \sum_{i=1}^{N_g} \sum_{j=1}^{N_g} [i + j - \mu_x(i) - \mu_y(j)]^3 \mathbf{P}(i,j)$	Measures the skewness of GLCM
	Contrast	$\text{Contrast} = \sum_{i=1}^{N_g} \sum_{j=1}^{N_g} \lvert i - j \rvert^2 \mathbf{P}(i,j)$	Measures of the local intensity variation of GLCM
	Correlation	$\text{Correlation} = \frac{\sum_{i=1}^{N_g} \sum_{j=1}^{N_g} ij\mathbf{P}(i,j) - \mu_i(i)\mu_j(j)}{\sigma_x(i)\sigma_y(j)}$	Measures gray tone linear-dependencies of the gray level
	Difference entropy	$\text{Difference entropy} = \sum_{i=0}^{N_g-1} \mathbf{P}_{x-y}(i) \log_2[P_{x-y}(i)]$	Measures entropy of processed GLCM matrix P_{X-Y}
	Dissimilarity	$\text{Dissimilarity} = \sum_{i=1}^{N_g} \sum_{j=1}^{N_g} \lvert i - j \rvert \mathbf{P}(i,j)$	Measures difference of entries in GLCM
	Energy	$\text{Energy} = \sum_{i=1}^{N_g} \sum_{j=1}^{N_g} [\mathbf{P}(i,j)]^2$	Measures of the homogeneity of GLCM
	Entropy	$\text{Entropy} = -\sum_{i=1}^{N_g} \sum_{j=1}^{N_g} \mathbf{P}(i,j) \log_2[\mathbf{P}(i,j)]$	Measures irregularity of GLCM
	Homogeneity1	$\text{Homogeneity1} = \sum_{i=1}^{N_g} \sum_{j=1}^{N_g} \frac{\mathbf{P}(i,j)}{1 + \lvert i - j \rvert}$	Measures closeness of GLCM
	Informational measure of correlation 1 (IMC1)	$\text{IMC1} = \text{HXY} - \frac{\text{HXY1}}{\max\{\text{HX}, \text{HY}\}}$	Secondary measure of homogeneity1

	Maximum probability	$\text{Maximum probability} = \max\{\mathbf{P}(i,j)\}$	Measures maximum value of GLCM matrix
	Sum average	$\sum_{i=2}^{2N_g}[i\mathbf{P}_{x+y}(i)]$	
	Sum variance	$\sum_{i=2}^{2N_g}(i-SE)^2\mathbf{P}_{x+y}(i)$	
	where $\mathbf{P}(i,j)$ denotes gray level co-occurrence matrix for $(\delta = 1, \alpha = 0)$,<br>N_g denotes number of discrete intensity values in the image,<br>N is the number of voxels in the ROI,<br>μ denotes mean of $\mathbf{P}(i,j)$,<br>$p_x(i) = \sum_{j=1}^{N_g}\mathbf{P}(i,j)$ denotes marginal row probabilities,<br>$p_y(i) = \sum_{i=1}^{N_g}\mathbf{P}(i,j)$ denotes marginal column probabilities,<br>μ_x represents the expected value of marginal row probability,<br>μ_y represents the expected value of marginal column probability,<br>σ_x denotes standard deviation of p_x,<br>σ_y denotes standard deviation of p_y,<br>$p_{x+y}(k) = \sum_{i=1}^{N_g}\sum_{j=1}^{N_g}\mathbf{P}(i,j)$, $i+j=k, k=2,3,\dots,2N_g$,<br>$p_{x-y}(k) = \sum_{i=1}^{N_g}\sum_{j=1}^{N_g}\mathbf{P}(i,j)$, $\mid i-j\mid = k, k = 0,1,\dots,N_g-1$,<br>$HX = -\sum_{i=1}^{N_g}\mathbf{P}_x(i)\log_2[p_x(i)]$ is the entropy of $\mathbf{P}_x$,<br>$HY = -\sum_{i=1}^{N_g}\mathbf{P}_y(i)\log_2[p_y(i)]$ is the entropy of $\mathbf{P}_y$,<br>$H = \sum_{i=1}^{N_g}\sum_{j=1}^{N_g}\mathbf{P}(i,j)\log_2[\mathbf{P}(i,j)]$ is the entropy of $\mathbf{P}(i,j)$<br>$HXY = -\sum_{i=1}^{N_g}\sum_{j=1}^{N_g}\mathbf{P}(i,j)\log_2[\mathbf{P}(i,j)]$ is the entropy of $\mathbf{P}(i,j)$<br>$HXY1 = -\sum_{i=1}^{N_g}\sum_{j=1}^{N_g}\mathbf{P}(i,j)\log(p_x(i)p_y(j))$.		
**Intensity size zone (ISZ, regional) features**	Size-zone variability	$\frac{1}{\Theta}\sum_{m=1}^{M}\left[\sum_{n=1}^{N}\boldsymbol{P}(m,n)\right]^2$	Measures variability in size of ROI
	Intensity variability	$\frac{1}{\Theta}\sum_{n=1}^{N}\left[\sum_{m=1}^{M}\boldsymbol{P}(m,n)\right]^2$	Measures variability in intensity of ROI
	where $\boldsymbol{P}(m,n)$ denotes intensity size zone matrix<br>Θ represents number of homogeneous areas in a tumor,<br>M denotes number of distinct intensity values,<br>N denotes size of homogeneous area in the matrix $\mathbf{P}(m,n)$		

Supplementary Table 2: Seventy-three clinicoradiological features used to predict alterations in targetable pathways and survival.

Category	Features
Clinical features	Sex
	Age
	Differentiation
	Tumor size
	T descriptor
	N descriptor
	M descriptor
	Tumor stage
	Smoking
	Smoking pack-year
Global variables	Mean
	Standard deviation
	Volume
	Density
	Mass
Histogram-based features	Variance
	Maximum
	Minimum
	Interquartile range (IQR)
	Range
	Root mean square (RMS)
	Skewness
	Kurtosis
	Energy
	Entropy
	Uniformity
	HU at the 2.5th percentile on histogram
	HU at the 25th percentile on histogram
	HU at the 50th percentile on histogram
	HU at the 75th percentile on histogram
	HU at the 97.5th percentile on histogram
Lung cancer-specific features	Mean value of positive pixels (MPP)
	Uniformity of distribution of positive gray level pixel values (UPP)
Shape features	Compactness 1
	Compactness 2

	Surface area<br>Maximum 3D diameter<br>Sphericity<br>Spherical disproportion<br>Surface to volume ratio (SVR)
Local features or gray level co-occurrence (GLCM, local features)	Autocorrelation<br>Cluster prominence<br>Cluster shade<br>Cluster tendency<br>Contrast<br>Correlation<br>Difference entropy<br>Dissimilarity<br>Energy<br>Entropy (H)<br>Homogeneity 1<br>Homogeneity 2<br>Informational measure of correlation (IMC)<br>Inverse difference normalized (IDN)<br>Maximum probability<br>Inverse variance<br>Sum average<br>Sum entropy<br>Sum variance<br>Variance
Regional features	Intensity variability<br>Size-zone variability
Emphysema features	Right emphysema index<br>Left emphysema index<br>Total emphysema index<br>Total emphysema volume<br>Right normal lung %<br>Left normal lung %<br>Total normal lung %<br>Normal lung volume<br>Right lung volume<br>Left lung volume<br>Total lung volume

Supplementary Table 3. Selected features for prediction of functional signaling pathways using univariate analysis,

	Redox stress		Differentiation		Apoptosis		Proliferation		Chromatic remodelers	
	Variables	*p* value	Variables	*p* value	Variables	*p* value	Variables	*p* value	Variables	*p* value
Clinical features			Smoking pack-years	0.100					Smoking pack-years	0.192
Global variables	Volume	0.159			Volume	0.067				
	Mass	0.163			Mass	0.066				
Histogram-based features	Maximum	0.180	Minimum	0.125	Maximum	0.113	HU at the 75th percentile	0.093	Energy	0.157
	Minimum	0.040	Energy	0.150	Minimum	0.187	HU at the 97.5th percentile	0.034		
	Range	0.101			Range	0.085				
	Energy	0.026			HU at the 2.5th percentile	0.128				
Lung cancer-specific features							MPP	0.021		
Shape features	Surface area	0.161	Spherical disproportion	0.200	Surface area	0.060				
	Maximum 3D diameter	0.112			Compactness 1	0.065				
					Maximum 3D diameter	0.056				
Local features	Autocorrelation	0.106			Autocorrelation	0.093	Cluster prominence	0.173	Difference entropy	0.172
	Cluster prominence	0.102			Cluster prominence	0.091	Cluster shade	0.191	Entropy	0.144
	Cluster shade	0.102			Cluster shade	0.090			Energy	0.125

	Cluster tendency	0.106			Cluster tendency	0.093			Homogeneity 1	0.174
	Correlation	0.133			Correlation	0.177			Homogeneity 2	0.188
	Sum average	0.114			Sum average	0.098			Maximum probability	0.107
	Variance	0.106			Variance	0.092			Inverse variance	0.185
Regional features	Size-zone variability	0.174	Intensity variability	0.190	Intensity variability	0.174				
	Intensity variability	0.190								
Emphysema features							Normal lung volume	0.018		
							Right lung volume	0.015		
							Left lung volume	0.042		
							Total lung volume	0.019		
